# Interdomain flip-flop motion visualized in flavocytochrome cellobiose dehydrogenase using high-speed atomic force microscopy during catalysis†
†Electronic supplementary information (ESI) available. See DOI: 10.1039/c7sc01672g



**DOI:** 10.1039/c7sc01672g

**Published:** 2017-08-03

**Authors:** Hirofumi Harada, Akira Onoda, Takayuki Uchihashi, Hiroki Watanabe, Naoki Sunagawa, Masahiro Samejima, Kiyohiko Igarashi, Takashi Hayashi

**Affiliations:** a Department of Applied Chemistry , Graduate School of Engineering , Osaka University , 2-1 Yamadaoka , Suita , Osaka 565-0871 , Japan . Email: onoda@chem.eng.osaka-u.ac.jp ; Email: thayashi@chem.eng.osaka-u.ac.jp; b Department of Physics , Nagoya University , Furo-cho, Chikusa-ku , Nagoya , 464-8602 , Japan . Email: uchihast@d.phys.nagoya-u.ac.jp; c Faculty of Natural Science and Technology , Kanazawa University , Kakuma , Kanazawa , 920-1192 , Japan; d Department of Biomaterials Sciences , Graduate School of Agricultural and Life Sciences , The University of Tokyo , Bunkyo-ku , 113-8657 , Japan . Email: aquarius@mail.ecc.u-tokyo.ac.jp; e VTT Technical Research Centre of Finland , P.O. Box 1000, Tietotie 2 , Espoo FI-02044 VTT , Finland

## Abstract

Cellobiose dehydrogenase (CDH) is a dual domain flavocytochrome, which consists of a dehydrogenase (DH) domain containing a flavin adenine dinucleotide and a cytochrome (CYT) domain containing *b*-type heme. To directly visualize the dynamic domain motion of class-I CDH from *Phanerochaete chrysosporium* (*Pc*CDH) during catalysis using high-speed atomic force microscopy, the apo-form of *Pc*CDH was anchored to a heme-immobilized flat gold surface that can specifically fix the orientation of the CYT domain. The two domains of CDH are found to be immobile in the absence of cellobiose, whereas the addition of cellobiose triggers an interdomain flip-flop motion involving domain–domain association and dissociation. Our results indicate that dynamic motion of a dual domain enzyme during catalysis induces efficient electron transfer to an external electron acceptor.

## Abstract 2

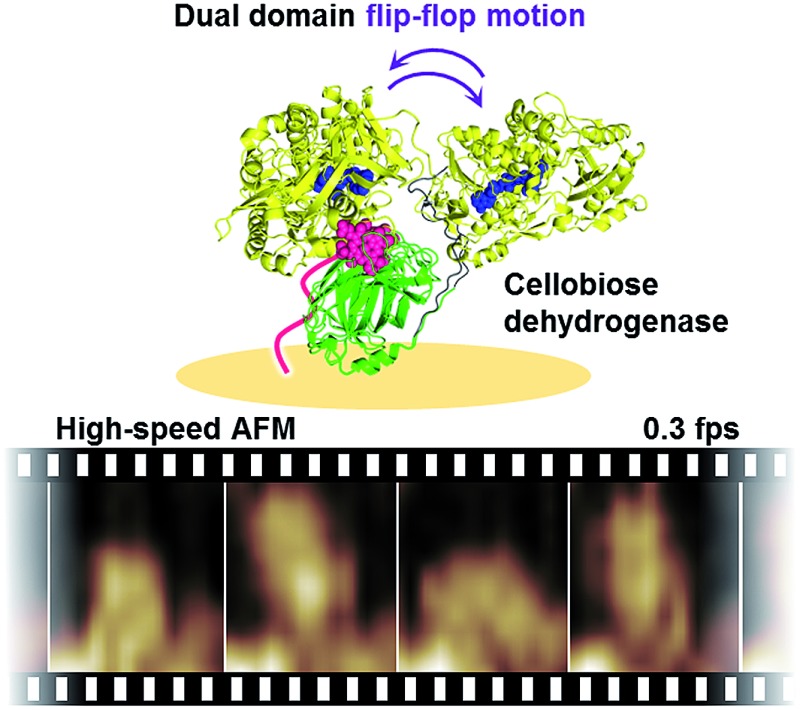
To visualize the dynamic domain motion of class-I CDH from *Phanerochaete chrysosporium* (*Pc*CDH) during catalysis using high-speed atomic force microscopy, the apo-form of *Pc*CDH was anchored to a heme-immobilized flat gold surface that can fix the orientation of the CYT domain.

## Introduction

Cellulose, a major component of plant cell walls, is a promising source of biomass, and conversions of recalcitrant cellulose into a plant-base small molecule are required to realize an ideal biorefinery process. Cellobiose dehydrogenases (CDHs),^1–4^ which are extracellular flavocytochromes, and copper-dependent lytic polysaccharide monooxygenases (LPMOs) have recently been found to contribute to a process of oxidative depolymerization of cellulose.^5–8^ In the process, electrons are donated from cellobiose *via* CDH to the copper site of LPMO and then activated LPMO catalyzes oxidative cleavage of glycosidic C–H bonds to boost the degradation of cellulose.^9–11^ Unraveling the mechanism of catalysis of cellobiose oxidation by CDH as an electron donor for LPMO therefore represents a focal point in terms of development of an ideal conversion process of cellulose.

**Fig. 1:**
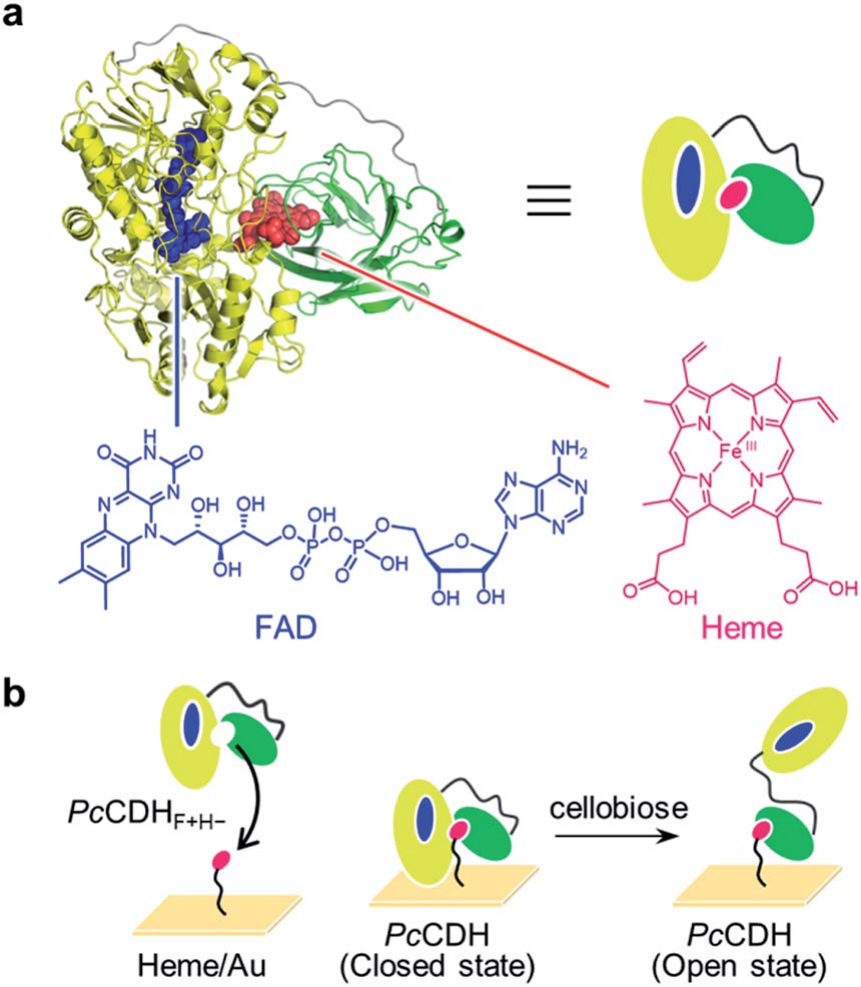
(a) *Pc*CDH consists of the FAD-binding DH domain in yellow (PDB: ; 1NAA) and the heme-binding CYT domain in green (PDB: ; 1D7C): the linker region, whose structure has not been solved, is illustrated as a gray line. Chemical structures of oxidized FAD and heme are shown. (b) Strategy for the immobilization of catalytically active *Pc*CDH on the heme-modified flat gold surface (heme/Au).

CDH, secreted by filamentous fungi from the phyla of Basidiomycota and Ascomycota, consists of two domains: a dehydrogenase (DH) domain containing flavin adenine dinucleotide (FAD) and a cytochrome (CYT) domain containing *b*-type heme (iron protoporphyrin IX).^12,13^ The domains are connected by a long flexible linker (Fig. 1a). The DH domain oxidizes the anomeric C1 position of cellobiose, and the two electrons carried by the reduced form of FAD in the DH domain are expected to be sequentially shuttled to heme *b* in the CYT domain *via* interdomain electron transfer (IET). The reduced form of CYT then donates the electrons to an external electron acceptor such as LPMO.^11,14,15^ Two electrons have been proposed to be transferred from the DH domain to the CYT domain through a ping-pong mechanism on the basis of previous kinetic studies.^16,17^ Two conformational snapshots of CDH in closed and open states are also determined by X-ray crystal structure analysis of full-length CDH. However, the closed and open structures are obtained from the different species, *Myriococcum thermophilum* (*Mt*CDH) and *Neurospora crassa* (*Nc*CDH), respectively (Fig. S1†).^18^ The next target is to demonstrate the single-molecule dynamic motion of CDH during catalysis.

We thus envisioned capturing the transient interdomain motion of CDH using high-speed atomic force microscopy (HS-AFM) to obtain a fundamental understanding of the structure and function of CDH. HS-AFM has recently been proven to be a powerful imaging technique capable of capturing dynamic motion of biomolecules temporally at the sub-second timescale and at sub-molecular lateral resolution.^19–23^ We herein report direct visualization of the dynamic motion of class-I CDH from the basidiomycete *Phanerochaete chrysosporium* (*Pc*CDH) by HS-AFM. To anchor and orient CDH on a flat surface for AFM imaging, we applied a technique for reconstitution of hemoproteins on a heme-immobilized surface *via* the heme–heme pocket interaction (Fig. 1b).^24–27^ The specific immobilization of the CYT domain of CDH on the flat surface enables us to directly visualize the interdomain motion of catalytically-active *Pc*CDH at a 300 ms temporal resolution.

## Results and discussion

To obtain high-resolution AFM images, we used deglycosylated *Pc*CDH, which has higher uniformity in terms of structure. Deglycosylated *Pc*CDH with characteristic absorption maxima at 380, 421, 450, 529, and 570 nm originating from FAD and ferric heme (hemin) was adjusted to pH 1.5 with an acidic solution to remove both cofactors (Fig. S2†). FAD was then incorporated into the resulting apoprotein *Pc*CDH_F–H–_ to afford the FAD-bound protein, *Pc*CDH_F+H–_.^28^ The reconstituted *Pc*CDH_F+H–_ has characteristic absorptions at 380 nm and 450 nm which are consistent with those observed for oxidized FAD, indicating incorporation of FAD into the DH domain of *Pc*CDH_F–H–_ (Fig. S3†). Upon addition of 1 equiv. of hemin into *Pc*CDH_F+H–_, the absorption at 420 nm was recovered. This result suggests that hemin can be incorporated into the CYT domain of *Pc*CDH_F+H–_ to afford the reconstituted protein, *Pc*CDH_F+H+_, in *ca.* 55% yield (Fig. S4†).

The reconstituted protein with both FAD and heme-*b* was found to retain the inherent activity of *Pc*CDH. The activity of *Pc*CDH_F+H+_ toward cellobiose oxidation was determined by reduction of dichloroindolindophenol (DCIP) or ferric cytochrome *c* as an oxidant according to the literature^29,30^ (Fig. S5†). The reduction of DCIP is mainly dependent on the redox reaction of the DH domain, whereas the reduction of cytochrome *c* depends mainly on the IET from FAD of the DH domain to hemin in the CYT domain. *Pc*CDH_F–H–_ completely lacks activity for reduction of both electron acceptors. *Pc*CDH_F+H–_ has activity for DCIP reduction and does not exhibit cytochrome *c* reduction activity due to the absence of the heme cofactor in the CYT domain. The reconstituted protein, *Pc*CDH_F+H+_ with both cofactors, has activity similar to *Pc*CDH in both assays. These results indicate that the step-by-step insertion of FAD and hemin converts *Pc*CDH_F–H–_ to catalytically active *Pc*CDH.

To construct *Pc*CDH_F+H+_ on a flat surface suitable for HS-AFM measurements, *Pc*CDH_F+H–_ was immobilized on a heme-modified gold surface (heme/Au) (Fig. S6 and S7†).^27,31–33^ We confirm the anchoring of *Pc*CDH_F+H–_ onto heme/Au by visualizing the process of protein reconstitution using HS-AFM (Fig. S8†). We first observed objects with a height of *ca.* 3 nm on heme/Au, which is consistent with the length of the 11-amino-1-undecathiol-linked heme (Fig. S7†). After addition of *Pc*CDH_F+H–_, larger spherical objects with a height of 6–8 nm were observed on the surface (Fig. S8†). The immobilization of *Pc*CDH_F+H–_ on the heme/Au surface was also confirmed by quartz crystal microbalance (QCM) measurements (Fig. S9†). The frequency decay of QCM was not observed upon addition of *Pc*CDH with the native heme cofactor to heme/Au. *Pc*CDH_F+H–_ with a vacant heme pocket showed a clear frequency decay. This finding indicates that *Pc*CDH_F+H–_ is incorporated *via* the specific interaction between the heme molecule on the gold surface and the heme pocket of the CYT domain, leading to oriented anchoring of the CYT domain of *Pc*CDH.

We tested the activity of *Pc*CDH anchored on the surface in the presence of cellobiose. After the *Pc*CDH@heme/Au substrate was immersed in a 50 μM cellobiose solution in NaOAc buffer (2 mM, pH 4.5), cellobiose and cellobino-1,5-lactone in the solution were analyzed by ESI-TOF MS. The conversion of cellobiose into cellobiono-1,5-lactone was confirmed under aerobic conditions, thereby indicating that *Pc*CDH linked on the gold surface exhibits the inherent enzymatic activity using O_2_ as an electron acceptor.

**Fig. 2:**
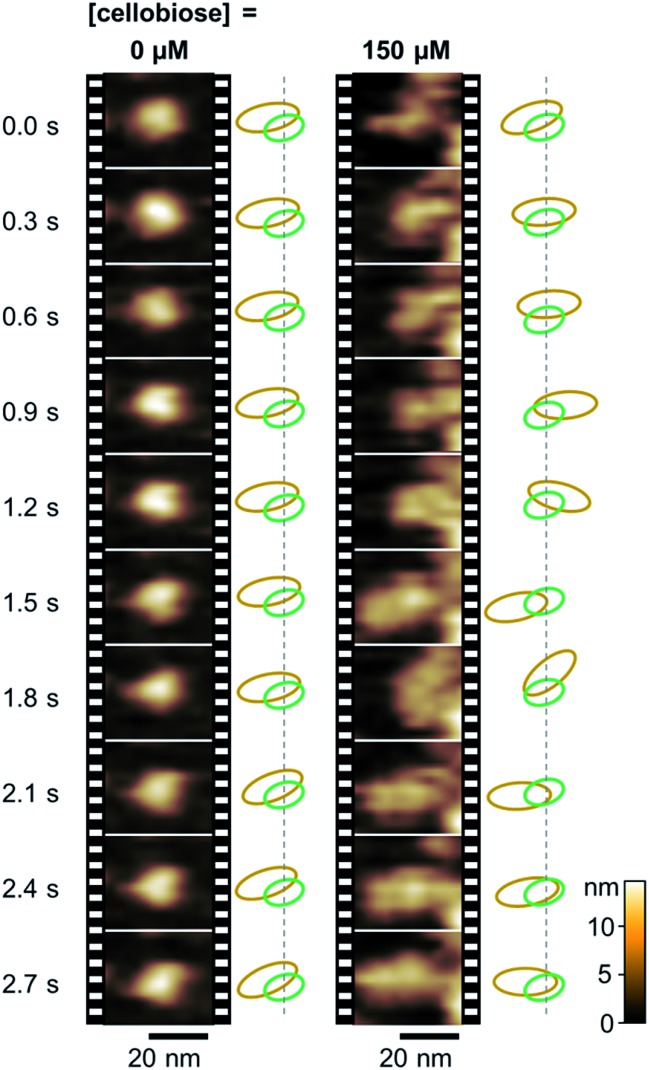
Real-time observation of *Pc*CDH anchored on the gold surface by HS-AFM. Time-evolution of the domain motion of *Pc*CDH in a different concentration of cellobiose (0 and 150 μM, Movies S1 and S4†). Estimated relative positions of the DH domain (orange) and the CYT domain (green) are illustrated on the right side of each set of image frames.

We then carried out real-time imaging of the domain motion of *Pc*CDH immobilized on the gold surface during the catalytic reaction using the HS-AFM technique. In the absence of cellobiose, immobilized *Pc*CDH is imaged as a round-shaped object with a height of 6–8 nm (Movie S1†). These motionless objects appear to correspond to the closed state of *Pc*CDH, as suggested by an X-ray crystal structure (Fig. S1†).^18^ Surprisingly, when cellobiose is added to the sample solution at the final concentration of 150 μM, these motionless objects of *Pc*CDH show a significant change in terms of motion (Fig. 2 and Movie S4†). Each *Pc*CDH contains a motionless part, which would be anchored at the same place on the surface, and a neighboring object moving rapidly around the motionless part. Considering that the CYT domain is anchored to the surface *via* the heme molecule, the moving part is identified as the DH domain of *Pc*CDH. In addition, we observed a change in the height of *Pc*CDH using a standard AFM arrangement (Fig. S10†). The average height of 6.6 ± 1.0 nm for *Pc*CDH in the absence of cellobiose was changed to 9.1 ± 0.9 nm in the presence of cellobiose. This is consistent with the heights estimated from the X-ray crystal structure of the closed state (*ca.* 6 nm) and the open state (*ca.* 10 nm) of *Pc*CDH. By contrast, the changes in the height profile were not detected by HS-AFM measurements. The DH domain, which is linked *via* a flexible linker, moves rapidly in the open state, and thus the height of the domain in rapid motion cannot be precisely measured using HS-AFM. The tip vibration would force the moving DH domain to remain near the surface in the fast scan mode.

**Fig. 3:**
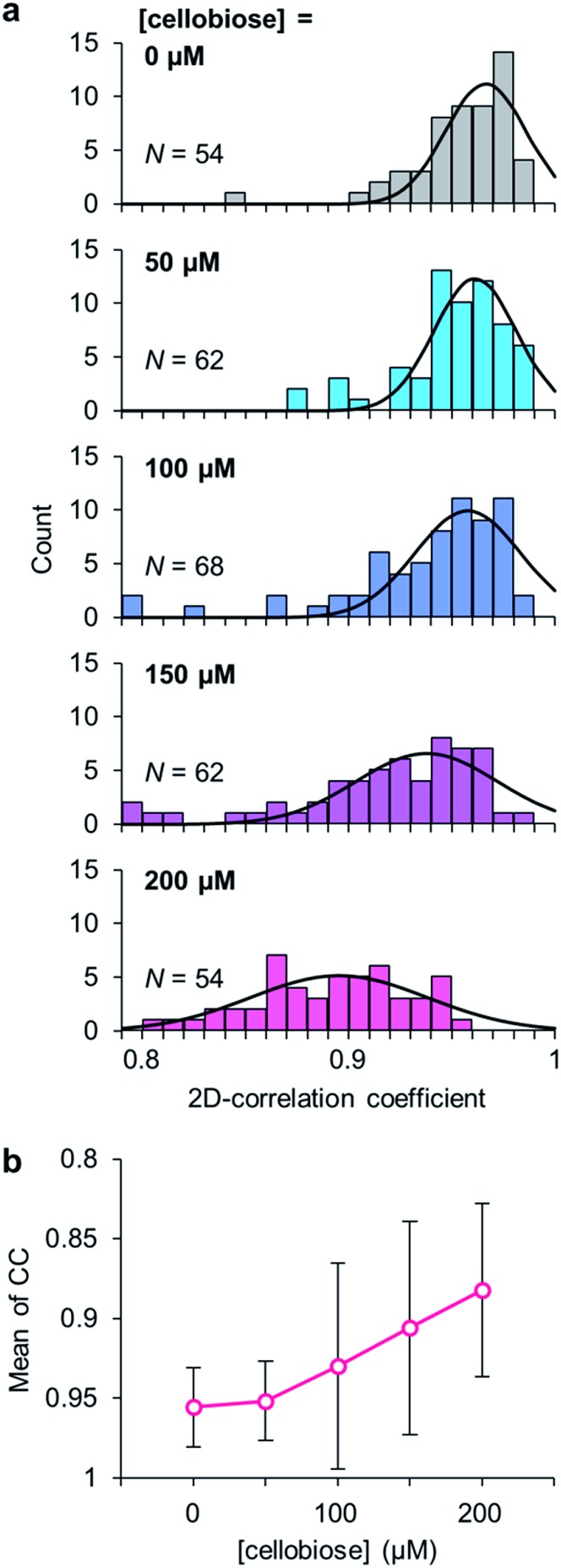
(a) Correlation coefficient (CC) histograms of *Pc*CDH at different concentrations of cellobiose (0, 50, 100, 150, and 200 μM). (b) The mean values of CC at different concentrations of cellobiose.

To clarify the evidence that the cellobiose molecule triggers the domain motion, the degree of the motion in *Pc*CDH was analyzed at different concentrations of cellobiose (Movies S2 to S5†). The conformational dynamics of *Pc*CDH were quantified using their two-dimensional correlation coefficient (CC) averaged through 300 ms × 250 frames for each molecule. The decrease in the CC value represents the difference between the two sequential images of the focused enzyme in the movie. In the absence of cellobiose, the CC value of *Pc*CDH is distributed around 0.97, suggesting the immobility of *Pc*CDH in the absence of the substrate. The CC values are gradually shifted from 0.96 to 0.88 as the concentration of cellobiose increases from 50 to 200 μM (Fig. 3). The decrease in the CC values indicates that the domain motion in *Pc*CDH becomes more intense. Furthermore, when cellobiose is removed from the observation cell by exchanging the buffer, the CC value shifts to 0.97, which is similar to that observed in the absence of cellobiose (Fig. S11 and Movie S6†). The lower shift of the CC distribution suggests the acceleration of the structural changes between the closed and the open states in *Pc*CDHs. Therefore, the degree of the motion in *Pc*CDH anchored on the gold surface increases in a cellobiose-dose-dependent manner, indicating that cellobiose binding followed by the reaction initiates the dynamic motion of the two domains.

**Fig. 4:**
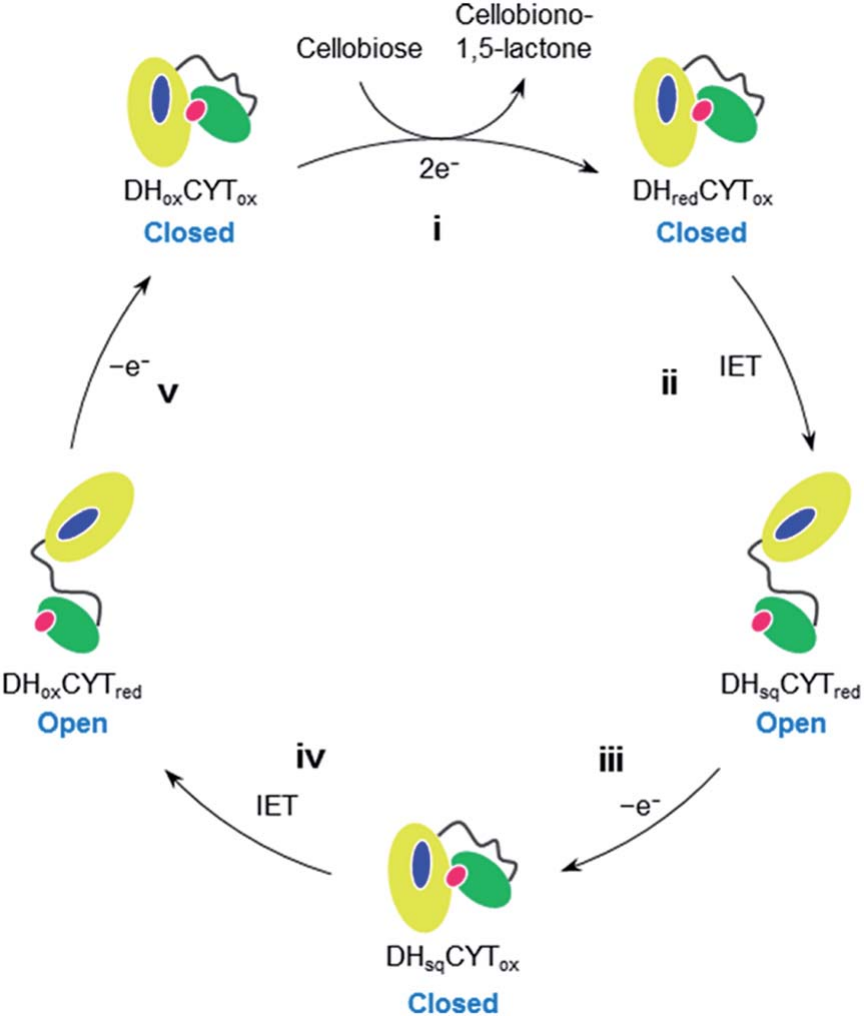
A proposed catalytic cycle of CDH including domain-flipping motions. (i) The resting form of CDH (DH_ox_CYT_ox_) binds cellobiose and then transfer of two-electrons from cellobiose to the FAD cofactor results in the generation of DH_red_CYT_ox_ and cellobiono-1,5-lactone as the product; (ii) one-electron IET from reduced FAD to ferric heme (DH_sq_CYT_red_) occurs, which has been proposed to represent the rate-limiting step in the cycle;^17,34,35^ (iii) the reduced CYT domain in DH_sq_CYT_red_ subsequently transfers one electron to an external electron acceptor, leading to the generation of DH_sq_CYT_ox_; (iv) second IET occurs from one-electron reduced FAD to ferric heme to produce DH_ox_CYT_red_; and (v) the reduced CYT domain transfers another electron to the external electron acceptor to regenerate the resting form.

In the oxidative depolymerization of cellulose, the DH domain of CDH is known to accept two electrons from cellobiose and then reduces the CYT domain through one-electron IET. The catalytic cycle of CDH through a ping-pong mechanism involving five steps has been proposed as shown in Fig. 4.^17,36^ *Pc*CDH is expected to be in the closed or open state when the heme is oxidized or reduced, respectively. The HS-AFM study for *Pc*CDH *in situ* gives us sequential views of the domain motions between the closed and open states during catalysis involving the cycle between the oxidation and reduction of the heme. The movement of the DH domain of *Pc*CDH is centered on the CYT domain during the cycle of the closed–open–closed states. The DH domain apparently moves back to similar positions approximately within 1 s, as estimated from the sequential HS-AFM images. The previous study using the small-angle X-ray scattering analysis indicates that in the presence of the inhibitor molecules, the conformational population of the open state increases relative to that of the closed state in both *Mt*CDH and *Nc*CDH.^18^ The small angle neutron scattering study revealed that glycosylated NcCDHIIA, which has a C-terminal carbohydrate binding module, has an extended conformation in the fully oxidized state.^37^ In the present study, we can directly observe single-molecule domain motion of deglycosylated *Pc*CDH triggered by binding of cellobiose. Therefore, the flip-flop domain motions of CDH appear to be strongly related to shuttling of electrons from cellobiose to a terminal electron acceptor.

## Conclusions

In this work, our data provide the first demonstration that *Pc*CDH can be immobilized on a flat gold surface *via* the CYT domain with a controlled orientation *via* the heme–heme pocket interaction and can still remain catalytically active toward cellobiose oxidation. In addition, this strategy enables the two domains of anchored *Pc*CDH to retain a degree of freedom in terms of interdomain motions required for the catalysis. As a result, we have directly visualized the interdomain flip-flop motions of *Pc*CDH triggered by the binding of cellobiose *in situ* using the HS-AFM technique. This observation implies that the flip-flop motion of the two domains in *Pc*CDH regulates the electron transfer process to the terminal electron acceptor, thereby harmonizing the entire electron relay through the enzymes in the oxidative process of cellulose depolymerization *in vivo*. The present method visualizing the domain motion of CDH on the flat surface by HS-AFM should be useful for investigating the dynamic motion of other multi-domain redox-active heme enzymes such as P450BM3.^38^ Our approach demonstrates that HS-AFM will provide novel insight into understanding the dynamic mechanism of enzyme catalysis including electron transfer as well as structural changes coupled with chemical reactions and photoactivation.^39^
