# Supplementary material for: Interdomain flip-flop motion visualized in flavocytochrome cellobiose dehydrogenase using high-speed atomic force microscopy during catalysis
Source: Chem Sci. 2017 Aug 3;8(9):6561–5. doi: 10.1039/c7sc01672g (PMC5627353; doi:10.1039/c7sc01672g)
Supplement: Supplementary file 1 [file SC-008-C7SC01672G-s001.pdf]

Electronic Supplementary Material (ESI) for Chemical Science.
This journal is © The Royal Society of Chemistry 2017

Supporting Information

Interdomain Flip-flop Motion Visualized in Flavocytochrome Cellobiose Dehydrogenase Using High-speed Atomic Force Microscopy during Catalysis.

Hirofumi Harada,[a] Akira Onoda,[a] Takayuki Uchihashi,*[b] Hiroki Watanabe,[c] Naoki Sunagawa,[d] Masahiro Samejima,[d] Kiyohiko Igarashi*[de] and Takashi Hayashi*[a]*

[a]Department of Applied Chemistry, Graduate School of Engineering, Osaka University, Suita, 565-0871, Japan.
[b]Department of Physics, Nagoya University, Furo-cho, Chikusa-ku, Nagoya 920-1192, Japan.
[c]Faculty of Natural Science and Technology, Kanazawa University, Kakuma, Kanazawa 920-1192, Japan.
[d]Department of Biomaterials Sciences, Graduate School of Agricultural and Life Sciences, The University of Tokyo, Bunkyo-ku, 113-8657, Japan.
[e]VTT Technical Research Centre of Finland, P.O.Box 1000, Tietotie 2, Espoo FI-02044 VTT, Finland.

E-mail: onoda@chem.eng.osaka-u.ac.jp, uchihast@d.phys.nagoya-u.ac.jp, aquarius@mail.ecc.u-tokyo.ac.jp, thayashi@chem.eng.osaka-u.ac.jp.

Table of Contents

1. Supplementary Method

2. Crystal Structures of *Mt*CDH and *Nc*CDH

3. UV/vis Absorption Spectra

4. Anchoring of *Pc*CDH

5. AFM Images of *Pc*CDH on Heme/Au

6. Correlation Coefficient Histograms of *Pc*CDH on Heme/Au

1. Supplementary Method

Reagents. All reagents were used without purification. Ultrapure water was demineralized using a Merck Milli-Q integral 3 system. Distilled ethanol was used for washing of gold electrodes.

Instruments. UV/vis absorption spectra were obtained on a Shimadzu UV-3150 or UV-2550 spectrophotometer equipped with a thermostatted cell holder (±0.1 °C), or on a Shimadzu BioSpec-nano spectrophotometer. The pH values were monitored with a Horiba F-52 pH meter. Purification of *Pc*CDH was performed using a GE Healthcare ÄKTA Purifier system at 4 °C. Quartz-crystal microbalance (QCM) measurements were carried out using AFFINIX $Q_N\mu$ (Initium, Japan). Electrochemical measurements were performed using a potentiostat (CompactStat, Ivium Technologies). ESI-TOF MS (electron spray ionization-time-of-flight-mass spectrometry) analyses were performed on a Bruker micrOTOF focus III mass spectrometer.

Illustrations. Protein structures were visualized using the program PyMOL (DeLano, www.pymol.org).

***Pc*CDH$_{F-H-}$.** *Pc*CDH$_{F-H-}$ was prepared according to an optimized version of a previously reported method.[S1] A solution of *Pc*CDH (3.5 μM) in 1 mL of 50 mM NaOAc buffer (pH 4.5) was added to 50 mM NaOAc buffer (pH 4.5) containing 3 M Gdn·HCl (guanidine hydrochloride) and 0.1 M histidine·HCl.

The solution was adjusted to pH 1.8 by careful addition of 5 M HCl_{aq} at 0 °C. Chilled 2-butanone (10 mL) was added to the *Pc*CDH solution on ice and the solution was gently mixed. The organic layer was removed after centrifugation at 3000 rpm for 10 min at 4 °C. The same procedure was repeated three times to completely remove the heme. The remaining 2-butanone dissolved in the aqueous phase was removed by dialysis (Wako, MWCO: 10 kDa) against 50 mM Tris·HCl (tris(hydroxymethyl)aminomethane hydrochloride) buffer (pH 8.0) containing 6 M Gdn·HCl and 200 mM NaCl for 12 h at 4 °C. The solution containing *Pc*CDH$_{F-H-}$ was further dialyzed against 50 mM NaOAc buffer (pH 4.5) three times. *Pc*CDH$_{F-H-}$ was then purified using a HiTrap Phenyl FF column (GE Healthcare) equilibrated with 50 mM NaOH (pH 4.5) containing 2 M $(NH_4)_2SO_4$ with a linear concentration gradient of $(NH_4)_2SO_4$. The purified *Pc*CDH$_{F-H-}$ was stored in the same buffer at −80 °C. The concentration of *Pc*CDH$_{F-H-}$ was determined by absorption at 280 nm (ε_{280} = 141 mM^{-1}•cm^{-1}).

***Pc*CDH$_{F+H-}$.** 2-mercaptoethanol (1.37 mg, 1.75 μmol (1 equiv. vs *Pc*CDH$_{F-H-}$)) was added to a solution of *Pc*CDH$_{F-H-}$ and the obtained solution was dialyzed against 6 M Gdn·HCl_{aq} in 50 mM

Tris·HCl buffer in the presence of 0.2 M NaCl for 5 h at 4 °C. The step gradient dialysis was performed with a different concentration of Gdn·HCl (4, 3, 2, and 1 M) in 50 mM Tris·HCl buffer (pH 8.0) containing 1 mM FAD for 12 h at 4 °C. *Pc*CDH_{F+H-} was refolded through the dialysis against three different Arg (arginine) buffers (Arg buffer = 400 mM Arg•HCl, 50 mM Tris·HCl (pH 8.0), 200 mM NaCl, 1 mM EDTA, 1 mM FAD). The protein was first dialyzed against Arg buffer containing 1 M Gdn•HCl, GSSG (glutathione disulfide) (1.07 mg, 1.75 μmol (1 equiv. vs *Pc*CDH_{F-H-})), and GSH (glutathione) (5.38 mg, 17.5 μmol (10 equiv. vs *Pc*CDH_{F-H-})) for 24 h at 4 °C. The protein was next dialyzed against Arg buffer containing 0.5 M Gdn•HCl for 12 h at 4 °C, and then Arg buffer for 6 h at 4 °C. The excess FAD was removed by dialysis against 50 mM NaOAc buffer (pH 4.5) three times and *Pc*CDH_{F+H-} was purified using a HiTrap Phenyl FF column using a procedure similar to the procedure used for purification of *Pc*CDH_{F-H-}. The purified *Pc*CDH_{F+H-} was stored at −80 °C. The concentration of FAD was calculated by the reported molar absorption coefficient (ε_{450} = 11.3 mM^{-1}•cm^{-1}).[S2]

***Pc*$\mathbf{CDH_{F+H+}}$.** A 2 μL aliquot of 0.25 mM hemin solution in 50 mM potassium phosphate buffer (pH 7.0) was added dropwise into 1.0 μM *Pc*CDH_{F+H-} in 3 mL of 50 mM NaOAc buffer (pH 4.5) on ice. The UV/vis absorption spectra were collected after equilibration for 30 min at 4 °C.

Activity assay. The DCIP (dichloroindolindophenol) and cytochrome *c* (cyt *c*) assays were carried out according to standard protocols reported in the literature with minor modifications.[S3] The activity was determined by following the absorption changes of DCIP at 520 nm (ε_{520} = 6.9 mM^{-1}•cm^{-1}) in 50 mM NaOAc (pH 4.5) containing 15 nM *Pc*CDH_{F-H-}, 600 μM DCIP, 7.5 mM cellobiose, 2 mM FAD, 2 mM heme, and 2 mM dithiothreitol at 25 °C. The activity was also determined by following the absorption changes of cyt *c* at 550 nm (ε_{550} = 19.6 mM^{-1}•cm^{-1}) in 50 mM NaOAc (pH 4.5) containing 13 nM *Pc*CDH_{F-H-}, 20 μM cyt *c*, 37.5 mM cellobiose, 2 mM FAD, 2 mM heme, and 5 mM dithiothreitol at 25 °C.

Preparation and characterization of the heme-modified gold surface (Heme/Au). Heme was coupled with a self-assembling monolayer (SAM) containing 6-hydroxy-1-hexanethiol and 11-amino-1-undecanethiol. An Au(111) surface on a mica (Phasis, Switzerland) was carefully cleaned with piranha solution (7:3 (v/v) of concentrated H_2SO_4 : 35% H_2O_2) for one minute, and thoroughly washed with deionized water. *Warning: Piranha solution is explosive and should be handled with extreme*

care; it is a strong oxidant and reacts violently with organic materials. All work should be performed in a fume hood. The electrodes were immersed in the solution of 1 mM mixed thiol (6-hydroxy-1-hexanethiol/11-amino-1-undecanethiol = 10,000/1) at 42 °C for 12 h. The electrode was washed with ethanol and dried under Ar flow (99.99%) to afford a SAM-modified gold surface (SAM/Au). SAM/Au was immersed in 2.5 mM hemin and 7.5 mM 1-ethyl-3-(3-dimethylaminopropyl)carbodiimide (EDC), and 3 mM triethylamine in DMSO (dimethylsulfoxide) (10 mL) and incubated in dark at 42 °C for 12 h. The surface was thoroughly washed with DMSO and ethanol, then dried under Ar flow.

The conjugation of heme on the gold surface was characterized by electrochemical measurements. Cyclic voltammograms were collected in a typical three-electrode configuration in 100 mM KPi (pH 7.0) as an electrolyte. An Ag|AgCl (saturated KCl_{aq}, +0.199 V vs. SHE) electrode and Pt-mesh electrode were employed as the reference electrode and counter electrode, respectively. The electrolyte was bubbled with Ar gas (99.99%) for 30 min at room temperature before each measurement. A limited area of the electrode sealed with a Teflon O-ring (Echo perfluor, ø = 7 mm, Taiwan Air Water Mach Co., Ltd.) was exposed to the electrolyte. The electrochemical analyses were carried out under an Ar atmosphere at 25 ºC. The surface concentration of the immobilized heme was estimated from the total current in the CV measurement.

Quartz-crystal microbalance (QCM) measurements. QCM measurements were performed using a cell equipped with a 27 MHz QCM plate oscillating at the fundamental frequency. The gold surface on the QCM sensor cell (ULVAC, gold substrate; ø = 2.5 mm) was modified with the heme moiety according to a similar procedure.[S4] The heme-immobilized surface was equilibrated with 200 µL of 50 mM NaOAc buffer (pH 4.5), and the frequency change was collected after the addition of a 0.3 µM *Pc*CDH and *Pc*CDH_{F+H-} in 50 mM NaOAc buffer (pH 4.5).

Standard Atomic Force Microscopy. The surface of *Pc*CDH@Heme/Au fabricated on glass plates was imaged using acoustic mode AFM with a SiN_4 tip (Olympus, AC40TS, tip apex; *d* = *ca.* 10 nm, spring constant: 0.1–0.2 Nm^{-1}) on an Asylum Research MFP-3DSA microscope. The images were recorded at a scan rate of 1 Hz with a resolution of 256 pixels per line. All AFM images were acquired in 50 mM NaOAc buffer (pH 4.5) at ambient temperature (24–26 °C).

ESI-TOF MS analysis of cellobino-1,5-lactone. The glassware was mounted on the *Pc*CDH@Heme/Au substrate sealed with a Teflon O-ring (Echo perfluor, ø = 7 mm, Taiwan Air Water Mach Co., Ltd.). After the *Pc*CDH@Heme/Au substrate was immersed in 50 µM cellobiose solution

in 150 μL of 2 mM NaOAc buffer (pH 4.5), cellobiose (m/z = 365.096 ± 0.002; calc. 365.105) and cellobino-1,5-lactone (m/z = 363.076 ± 0.020; calc. 363.090) in the solution were analyzed by ESI-TOF MS. We tested the activity of *Pc*CDH anchored on the surface in the presence of cellobiose. The conversion of cellobiose into cellobiono-1,5-lactone was confirmed by ESI-TOF MS, indicating that *Pc*CDH linked on the gold surface exhibits the inherent enzymatic activity.

2. Crystal Structures of *Mt*CDH and *Nc*CDH

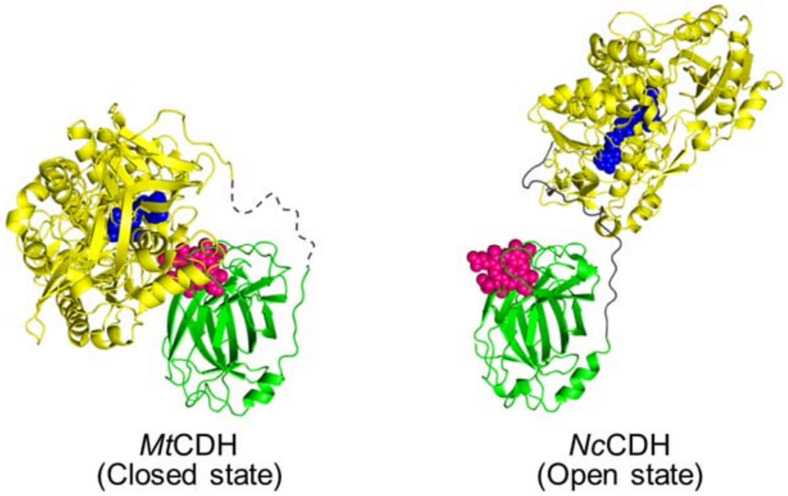

Figure S1. Crystal structures of *Mt*CDH in the closed state (PDB: 4QI6) and *Nc*CDH in the open state (PDB: 4QI7).

3. **UV/vis Absorption Spectra**

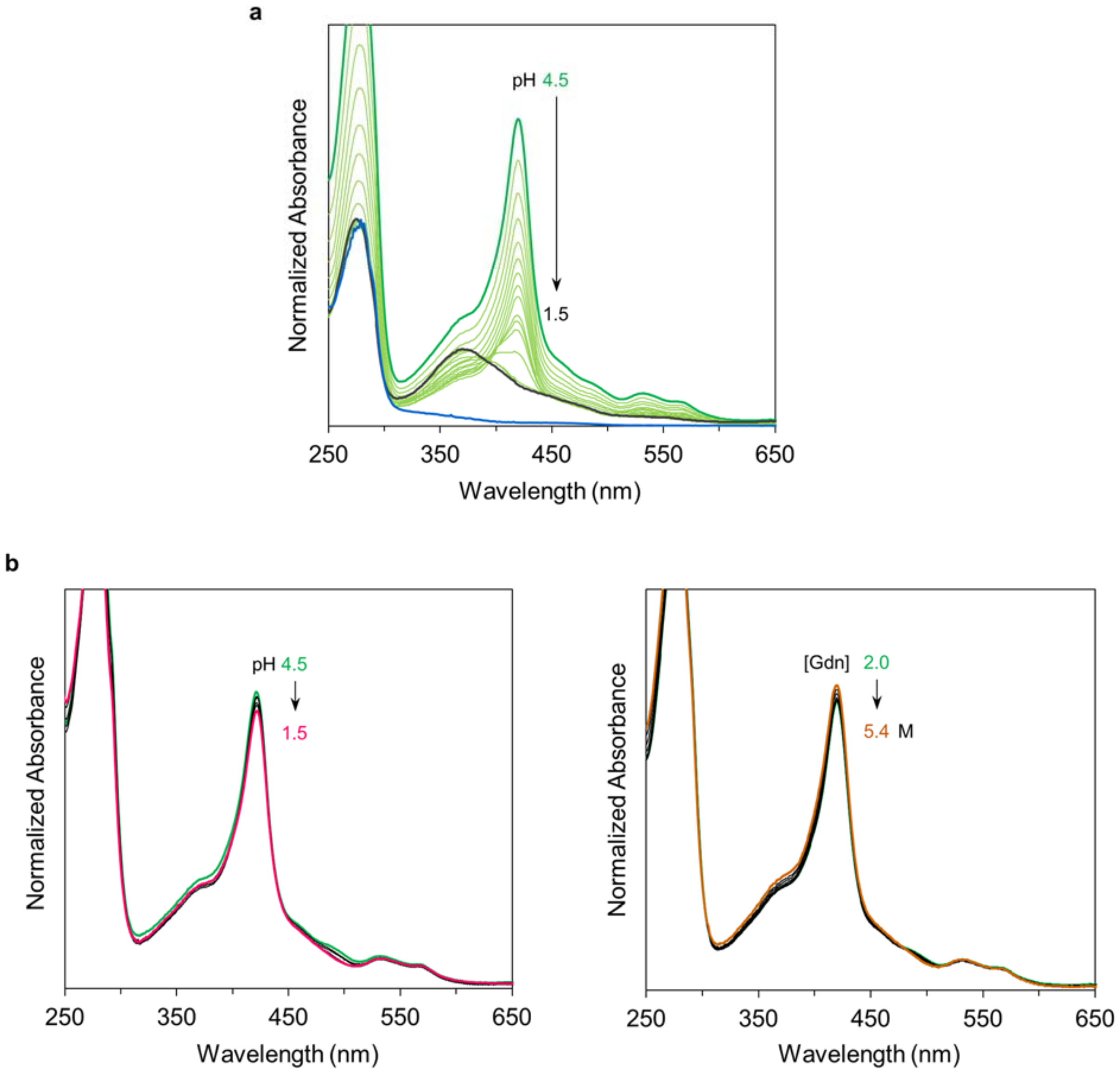

Figure S2. UV/vis absorption spectra of *Pc*CDH during removal of cofactors. (a) UV/vis absorption spectra of *Pc*CDH at different pH values from pH 4.5 (green) to pH 1.5 (black) in the presence of 3 M Gdn·HCl_{aq}. The characteristic Soret and Q absorption bands at 421, 529, and 570 nm of hemin incorporated within the pocket of CYT clearly disappear with the concomitant increase of the absorption maxima at 370 nm, indicating the removal of heme from *Pc*CYT. Purified *Pc*CDH_{F-H-} does not exhibit the absorptions of heme and FAD (blue line). (b) Both cofactors cannot be removed by acid (pH 4.5−1.5) or denaturing reagent Gdn·HCl within the concentration range of 2.0−5.4 M.

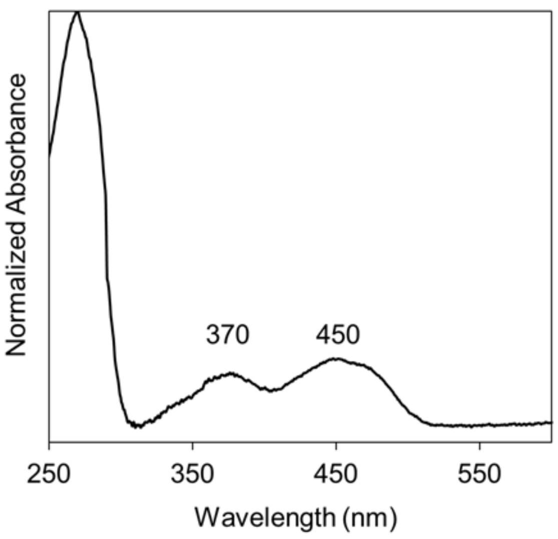

Figure S3. UV/vis absorption spectra of *Pc*CDH$_{F+H-}$. FAD was inserted into *Pc*CDH$_{F-H-}$ in the presence of 2-mercaptoethanol and oxidized glutathione during a step gradient dialysis of Gdn·HCl. The spectrum was obtained in 25 mM Tris·HCl buffer (pH 8.0).

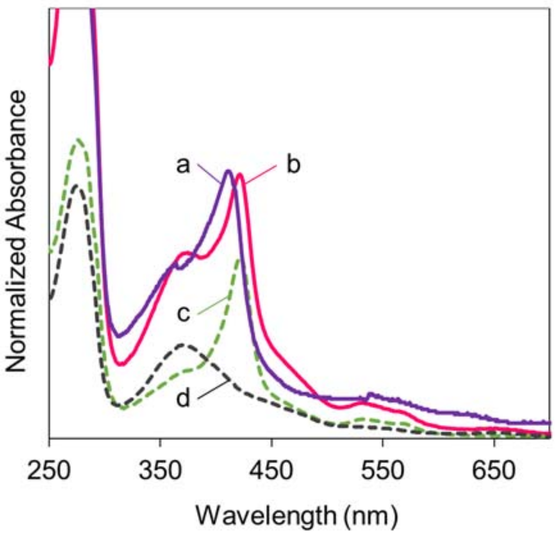

Figure S4. Reconstitution of *Pc*CDH$_{F+H-}$ with heme cofactor. (a) UV/vis absorption spectra of *Pc*CDH$_{F-H-}$ after the addition of 1 equiv. of hemin. (b) The simulated spectrum, which represents the summation of (c) *Pc*CDH (55%) and (d) free-heme + *Pc*CDH$_{F-H-}$ (45%).

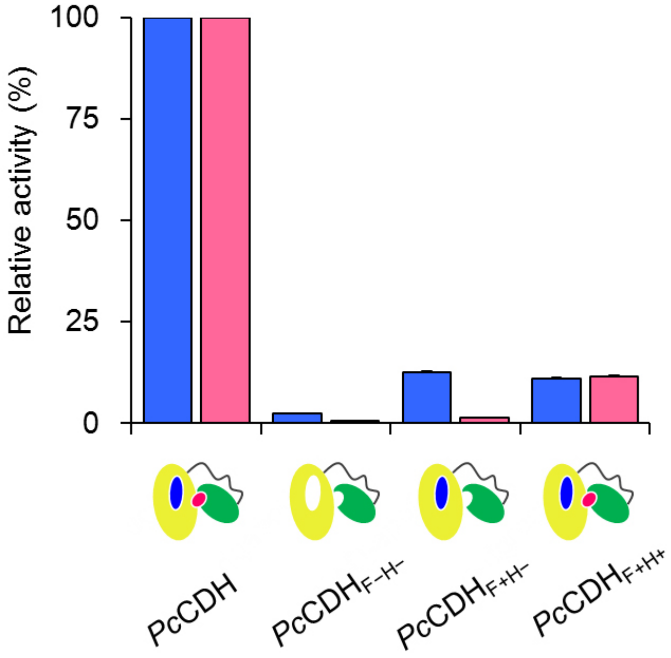

Figure S5. Catalytic activity of reconstituted *Pc*CDH toward oxidation of cellobiose. The activity determined by DCIP (dichloroindolindophenol) reduction (blue) and cytochrome *c* reduction (red) for *Pc*CDH, *Pc*CDH$_{F-H-}$, *Pc*CDH$_{F+H-}$, and *Pc*CDH$_{F+H+}$. DCIP is a two-electron acceptor for the DH domain. Cytochrome *c* is a one-electron acceptor for the CYT domain. Reduced FAD and heme without apoprotein showed no activity for both assays. The specific activities of *Pc*CDH, *Pc*CDH$_{F+H-}$, and *Pc*CDH$_{F+H+}$ are 25±1.6, 3.2±0.1, and 2.8±0.1 (µmol•min^{-1}•mg^{-1}), respectively.

4. Anchoring of *Pc*CDH

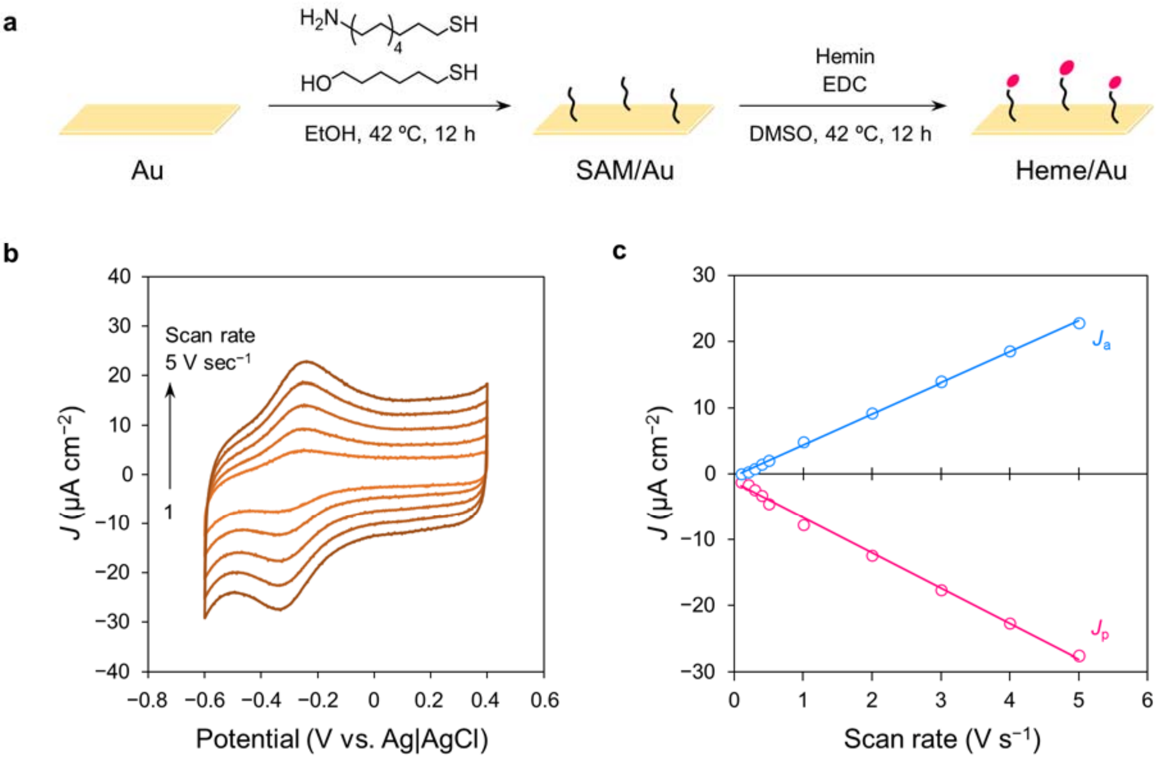

Figure S6. Electrochemical characterization of Heme/Au. (a) Scheme for preparation of Heme/Au. (b) Cyclic voltammograms of Heme/Au in 100 mM KPi (pH 7.0). An Fe^{II}/Fe^{III} redox couple of heme was observed at approximately −0.3 V (vs. Ag|AgCl). (c) Plots of the anodic and cathodic peak currents (J_a and J_c) versus scan rates. Both J_a and J_p are proportionate to the scan rate, indicating that the redox-active heme is immobilized on the gold surface. The surface concentration of the immobilized heme was estimated to be 1.3 ± 0.4 pmol·cm^{-2}, as determined by an electrochemical analysis of the Fe^{II}/Fe^{III} redox couple of the heme. The cathodic and anodic peak currents were determined unequivocally with the conventional background correction process using a flat portion of the voltammogram to subtract the non-faradaic current.

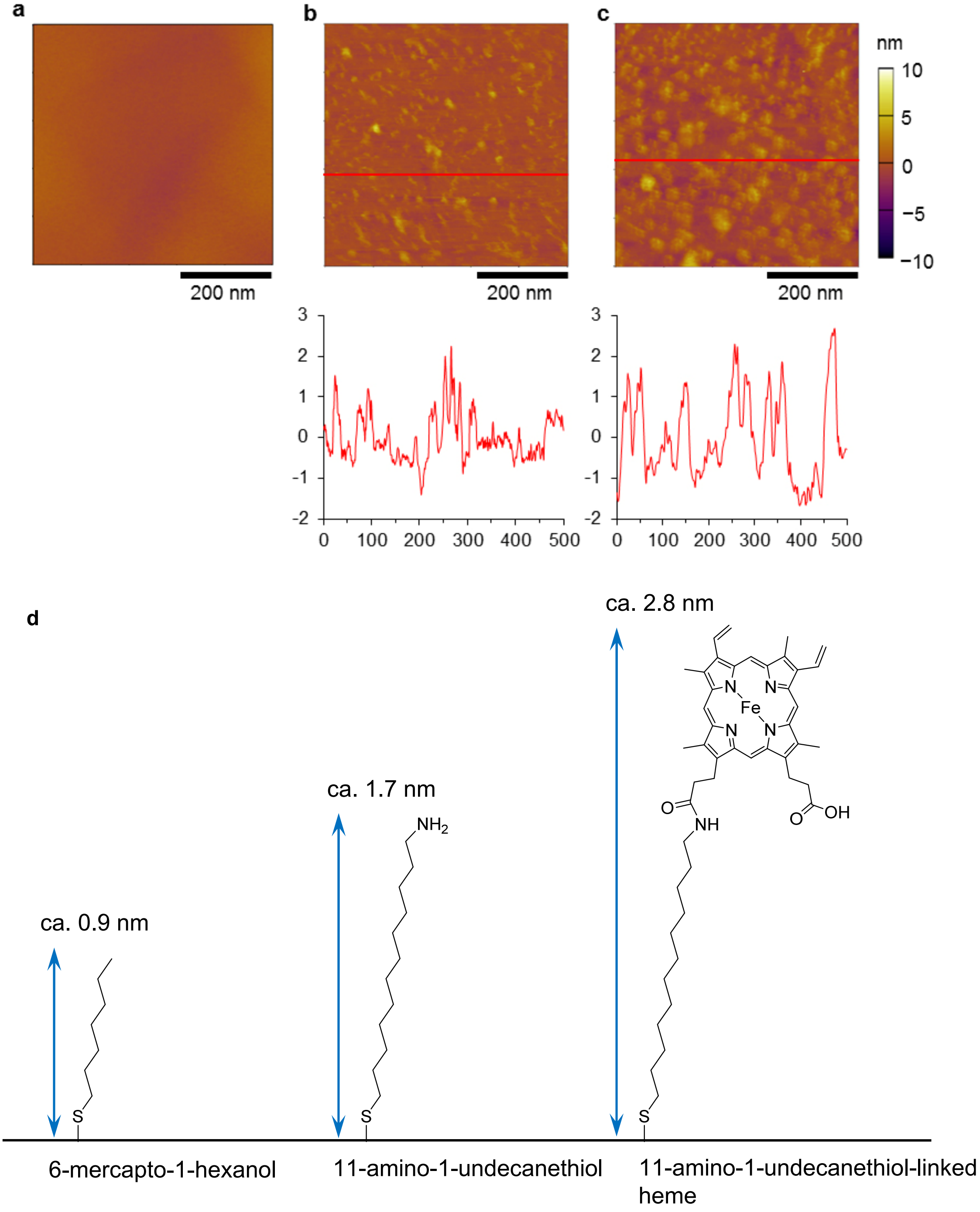

Figure S7. AFM images of (**a**) Au, (**b**) SAM/Au, and (**c**) Heme/Au obtained using a standard AFM setup. Height traces are shown below the images. Samples were prepared using an Au(111) surface annealed on mica. The SAM was constructed using a mixture of 6-hydroxy-1-hexanethiol and 11-amino-1-undecanethiol (10,000 : 1 molar ratio). Roughness factors, arithmetic average of the absolute values (R_a) and the root mean square roughness values (R_q) are determined as follows: 0.45 nm and 0.56 nm for SAM/Au; 0.86 nm and 1.02 nm for Heme/Au. (**d**) Chemical structures and the size of the surface molecules of 6-hydroxy-1-hexanethiol, 11-amino-1-undecanethiol 11-amino-1-undecathiol-linked heme.

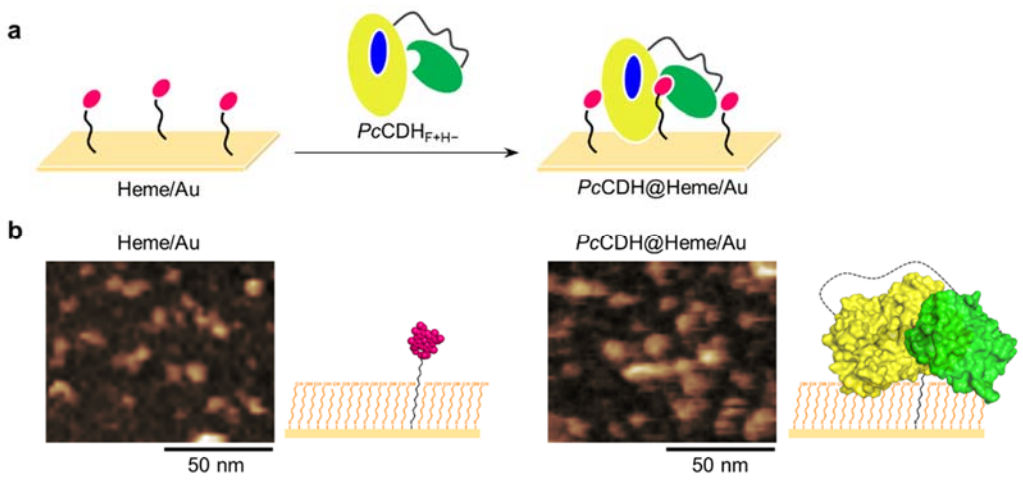

Figure S8. *Pc*CDH$_{F+H-}$ immobilized on a heme-modified gold surface to yield *Pc*CDH$_{F+H+}$. (a) Scheme for immobilizing *Pc*CDH$_{F+H-}$ on a SAM on a gold surface tethering heme moieties via a heme−heme pocket interaction. (**b**) HS-AFM images for heme-modified Heme/Au (left) and protein-linked Heme/Au in the presence of 0.14 µM *Pc*CDH$_{F+H-}$ (right) measured in NaOAc buffer (pH 4.5). The height range of the spherical objects is approximately 6−8 nm. Cross-sectional illustrations of the heme-modified gold surface (height of *ca.* 3 nm), and *Pc*CDH-modified gold surface (height of *ca.* 6 nm) are provided beside the images.

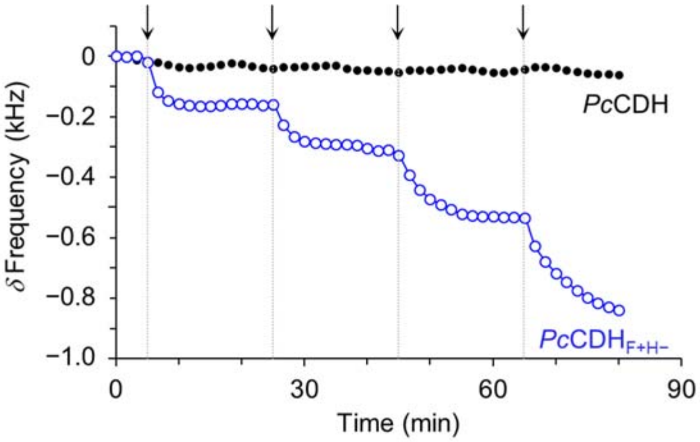

Figure S9. QCM measurement during the immobilization of *Pc*CDH$_{F+H-}$ on Heme/Au. Frequency changes in response to the addition of *Pc*CDH$_{F+H-}$ (blue) and *Pc*CDH (black) in QCM measured in 50 mM NaOAc buffer (pH 4.5) at 20 °C. Arrows represent the addition of the sample solution.

5. AFM Images of *Pc*CDH on Heme/Au

High-Speed Atomic Force Microscopy (HS-AFM) apparatus. HS-AFM images were recorded with a custom-designed HS-AFM apparatus.[S5] The dimensions of the cantilever (Olympus) are 6−7 μm length, 2 μm width, and 90 nm thickness. The nominal spring constant is 0.1−0.2 N/m, the resonant frequency was 0.7−1 MHz, and the quality factor in aqueous solution was *ca.* 2. For AFM imaging, the free oscillation amplitude was set to 1−2 nm, and the set-point amplitude was approximately 90% of the free oscillation amplitude. An amorphous carbon tip was grown on the original tip through electron-beam deposition to provide a sharp probe. The tip length was adjusted to *ca.* 1 μm. The tip was sharpened by plasma etching under Ar gas (tip apex, *d* = *ca.* 4 nm).

HS-AFM imaging and processing. In the HS-AFM imaging, Au(111) thin film on mica (Phasis, Switzerland) was used for the preparation of Heme/Au (See Figure S7). Heme/Au was added to the sample stage of HS-AFM, after 200 μL of 50 mM NaOAc buffer (pH 4.5) was added to the entire surface of the sample. The solution (10 μL) of 1 μM $Pc\mathrm{CDH}_{\mathrm{F+H-}}$ was added to the Heme/Au substrate to provide a final concentration of 50 nM. The concentration of cellobiose was adjusted by adding 2−8 μL of 5 mM cellobiose in 50 mM NaOAc buffer (pH 4.5). An aliquot of the same volume was removed to maintain the total buffer volume. A scan area of *ca*. 45 × 22 nm^2 with (100−150) × (50−75) pixels was typically used. The AFM images were captured at frame rates of 3.3 fps at room temperature. After capturing images, we tracked a target molecule using two-dimensional (2D) correlation analysis to compensate for the slow drift of the sample stage position in the x- and y-directions. All images were processed by a line-wise correction with histogram alignment, which removes accidental string noise along the x-scanning direction and the pixel sizes of images were increased by pixel-pixel spline interpolation using SPIP software (Image Metrology, Denmark).

Correlation analysis. To determine the time evolution of the conformational changes of the two domains in *Pc*CDH, a 2D correlation coefficient (*CC*) for temporally adjacent frames was calculated using custom-programmed software with the following method. A region of interest (ROI) was arbitrarily chosen so that the ROI in the reference frame fully contains *Pc*CDH. The *CC* is defined as,

$$\mathrm{CC} = \Sigma_m \Sigma_n (H_{mn} - H_a)(R_{mn} - R_a)/((\Sigma_m \Sigma_n (H_{mn} - H_a)^2) (\Sigma_m \Sigma_n (R_{mn} - R_a)^2))^{1/2}$$

Here, H_{mn} and R_{mn} represent heights at pixel points (*m*, *n*) in a to-be-analyzed ROI and a reference ROI of the reference frame, respectively. We assigned the same frame in the last image for the reference ROI. H_a and R_a are mean values of the height matrices *H* and *R*, respectively. The *CC* value was calculated throughout 300 ms/frame × 250 images for the single ROI. The set of the *CC* values (249 points) for the single ROI was averaged for this period of time, 300 ms/frame × 250, to calculate the

averaged *CC* value for the single ROI. Approximately 60 ROI values were analyzed for each condition. Histograms were generated using the mean of *CC* values of each ROI.

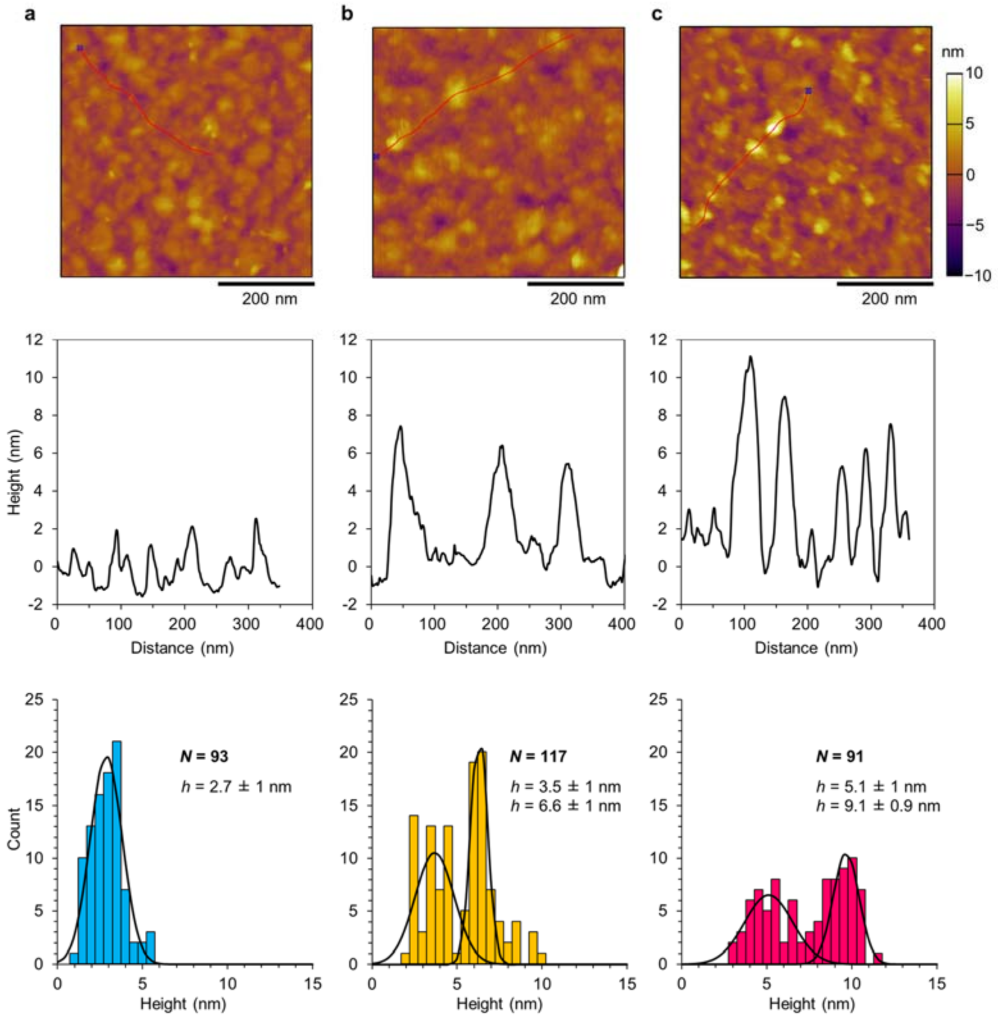

Figure S10. Standard AFM images *Pc*CDHs on Heme/Au. The surfaces of (**a**) Heme/Au, (**b**) *Pc*CDH@Heme/Au in the absence of cellobiose, and (**c**) *Pc*CDH@Heme/Au in the presence of cellobiose (5 mM) were analyzed in 50 mM NaOAc buffer (pH 4.5). The peaks in the image were randomly collected and the height distribution was plotted as a histogram. The plots were fitted with a Gaussian distribution to calculate the height average. The histograms for *Pc*CDH@Heme/Au in the absence and presence of cellobiose exhibit two apparent distributions. We assigned the greater height average within the two distributions to the height of *Pc*CDH. The average height of 6.6 ± 1.0 nm for *Pc*CDH in the absence of cellobiose was changed to 9.1 ± 0.9 nm in the presence of cellobiose. This is consistent with the heights estimated from the X-ray crystal structure of the closed state (*ca.* 6 nm) and the open state (*ca.* 10 nm) of *Pc*CDH.

6. Correlation Coefficient Histograms of *Pc*CDH on Heme/Au

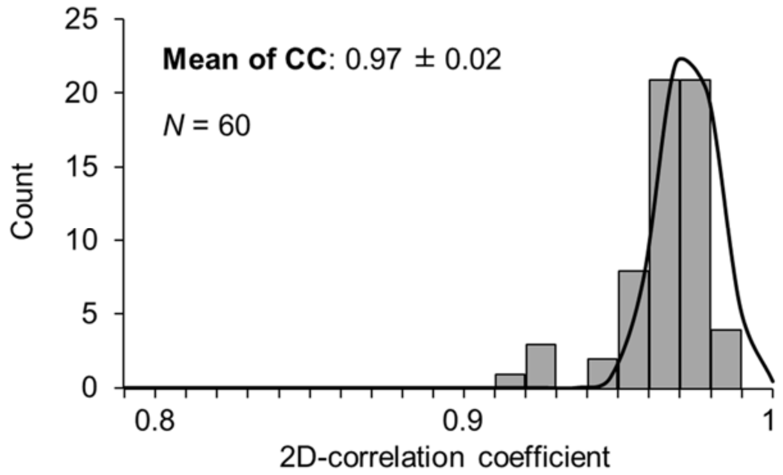

Figure S11. Correlation coefficient histograms of *Pc*CDH@Heme/Au in the absence of cellobiose. The 2D correlation coefficients (CCs) were collected using the images of Supplementary Movie 6. The mean CC value of 0.97 suggests that *Pc*CDH is immobile after the removal of cellobiose.

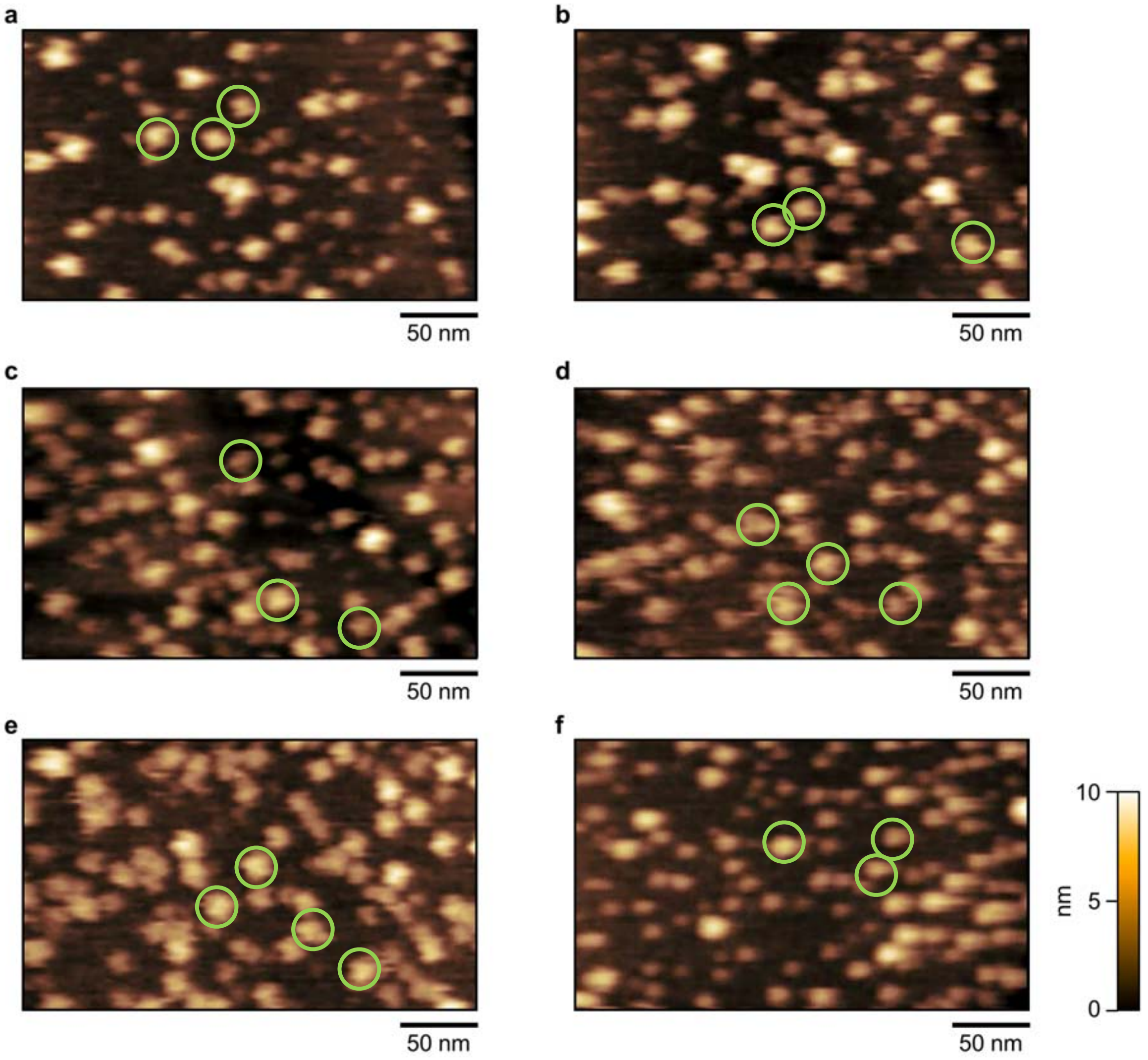

Figure S12. HS-AFM images taken from (**a**) Movie S1, (**b**) Movie S2, (**c**) Movie S3, (**d**) Movie S4, (**e**) Movie S5, and (**f**) Movie S6. The objects of interest in the movies are labeled in green circles.

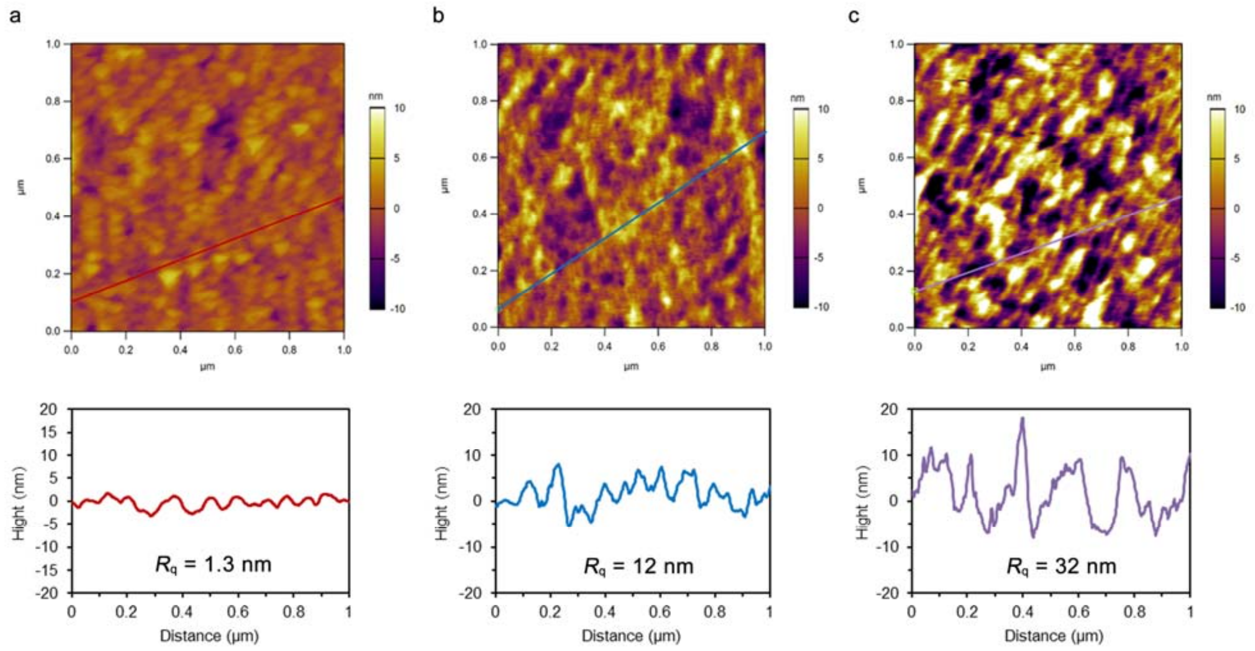

Figure S13. Standard AFM images of glycosylated *Pc*CDHs (g*Pc*CDHs) on Heme/Au. The surfaces of (a) Heme/Au, (b) g*Pc*CDH@Heme/Au in the absence of cellobiose, and (c) g*Pc*CDH@Heme/Au in the presence of cellobiose (5 mM) were analyzed in 50 mM NaOAc buffer (pH 4.5). Height traces are shown below the images.

Captions for Supporting Online Movies

Movie S1. HS-AFM images of *Pc*CDH@Heme/Au without cellobiose. x/y = 3 00/180 nm, 4.52x playback, total time 75 s. Scale bar and height lookup table (LUT) of the images are shown in Figure 12.

Movie S2. HS-AFM images of *Pc*CDH@Heme/Au with cellobiose (final conc. 50 μM). x/y = 300/180 nm, 4.52x playback, total time 75 sec. Scale bar and height LUT of the images are shown in Figure 12.

Movie S3. HS-AFM images of *Pc*CDH@Heme/Au with cellobiose (final conc. 100 μM). x/y =300/180 nm, 4.52x playback, total time 75 sec. Scale bar and height LUT of the images are shown in Figure 12.

Movie S4. HS-AFM images of *Pc*CDH@Heme/Au with cellobiose (final conc. 150 μM). x/y = 300/180 nm, 4.52x playback, total time 75 sec. Scale bar and height LUT of the images are shown in Figure 12.

Movie S5. HS-AFM images of *Pc*CDH@Heme/Au with cellobiose (final conc. 200 μM). x/y = 300/180 nm, 4.52x playback, total time 75 sec. Scale bar and height LUT of the images are shown in Figure 12.

Movie S6. HS-AFM images of *Pc*CDH@Heme/Au after removal of cellobiose in observation cell by exchanging the buffer. x/y = 300/180 nm, 4.52x playback, total time 75 sec. Scale bar and height LUT of the images are shown in Fig. 12.

References

(1) Teale, F. W. J. *Biochim. Biophys. Acta* **1959**, *35*, 289−291.

(2) Bao, W. J.; Usha, S. N.; Renganathan, V. *Arch. Biochem. Biophys.* **1993**, *300*, 705−713.

(3) (a) K. Igarashi, M. F. J. M. Verhagen, M. Samejima, M. Schülein, K.-E. L. Eriksson and T. Nishino, *J. Biol. Chem.*, 1999, **274**, 3338−3344. (b) Henriksson, G.; Sild, V.; Szabó, I. J.; Pettersson, G.; Johansson, G. *Biochim. Biophys. Acta* **1998**, *1383*, 48−54.

(4) Onoda, A.; Kakikura, Y.; Uematsu, T.; Kuwabata, S.; Hayashi, T. *Angew. Chem. Int. Ed.* **2012**, *51*, 2628−2631.

(5) (a) Ando, T.; Kodera, N.; Takai, E.; Maruyama, D.; Saito, K.; Toda, A. *Proc. Natl Acad. Sci. USA* **2001**, *98*, 12468−12472. (b) Ando, T.; Uchihashi, T.; Fukuma, T. *Prog. Surf. Sci.* **2008**, *83*, 337−437.
